# A twenty-year dataset of high-resolution maize distribution in China

**DOI:** 10.1038/s41597-023-02573-6

**Published:** 2023-09-26

**Authors:** Qiongyan Peng, Ruoque Shen, Xiangqian Li, Tao Ye, Jie Dong, Yangyang Fu, Wenping Yuan

**Affiliations:** 1 International Research Center of Big Data for Sustainable Development Goals, School of Atmospheric Sciences, Sun Yat-sen University, Zhuhai, 519082 Guangdong China; 2 Faculty of Geographical Science, Beijing Normal University, Beijing, 100875 China; 3 College of Geomatics & Municipal Engineering, Zhejiang University of Water Resources and Electric Power, Hangzhou, 310018 Zhejiang China

**Keywords:** Environmental impact, Agriculture

## Abstract

China is the world’s second-largest maize producer, contributing 23% to global production and playing a crucial role in stabilizing the global maize supply. Therefore, accurately mapping the maize distribution in China is of great significance for regional and global food security and international cereals trade. However, it still lacks a long-term maize distribution dataset with fine spatial resolution, because the existing high spatial resolution satellite datasets suffer from data gaps caused by cloud cover, especially in humid and cloudy regions. This study aimed to produce a long-term, high-resolution maize distribution map for China (China Crop Dataset–Maize, CCD-Maize) identifying maize in 22 provinces and municipalities from 2001 to 2020. The map was produced using a high spatiotemporal resolution fused dataset and a phenology-based method called Time-Weighted Dynamic Time Warping. A validation based on 54,281 field survey samples with a 30-m resolution showed that the average user’s accuracy and producer’s accuracy of CCD-Maize were 77.32% and 80.98%, respectively, and the overall accuracy was 80.06% over all 22 provinces.

## Background & Summary

Food security is the foundation of human survival and national security. According to the Food and Agriculture Organization (FAO), nearly 10% of the world’s population suffered from hunger in 2020^1^. With the rising global population, global food demand will increase by 100–110% in 2050 compared to 2005^2^, which may significantly challenge food security^3,4^. Maize is one of the most widely planted cereals in the world, and sustainable maize production plays a crucial role in meeting the rapidly growing food demand and ensuring national and global food security. Maize represents an important source of food for humans and animals and industrial raw material^5,6^. Since 2001, maize has surpassed rice as the world’s second-largest cereal^1^. In 2019, maize accounted for about 12% of global crop production^1^.

Large-scale and long-term distribution maps of maize are essential for maintaining food security and achieving sustainable development^7,8^. Distribution maps of maize are not only crucial data for simulating maize yield^9^, but also the basis for identifying maize phenology^10,11^. Additionally, a distribution map of maize can be used as a reference for predicting future maize distributions by exploring the driving forces of crop distribution patterns^12^. Because of the use of fertilizers such as nitrogen, a large amount of long-term existed greenhouse gas N_2_O is associated with the maize planting process^13,14^. Therefore, the distribution map of maize can also be an important source of data for simulating greenhouse gas emissions from agricultural ecosystems and plays an important role in determining the regional budget for greenhouse gases^15,16^. In addition, compared with C_3_ plants, a C_4_ crop type such as maize has stronger photosynthetic capacity^17^. To accurately estimate the global crop gross primary productivity, mapping the long-term spatial distribution of maize is necessary^18^.

As the world’s second-largest maize producer, China produced 260.95 million tons of maize in 2019, accounting for 22.72% of global maize production^1^. However, in recent years, climate change and extreme weather events have profoundly affected China’s agricultural production^19–21^. Specifically, maize can be cultivated in various geographical regions and environmental conditions, including large areas with no irrigation^22^, and is highly sensitive to climate change. Studies have shown that from 1979 to 2016, for every 1 °C increase in temperature, China’s maize yield decreased by 1.7%^23^. In addition, Yuan *et al*.^24^ found that the planting area of maize in China has significantly increased with the improvement of economic returns, leading to substantial changes in its spatial distribution pattern. Therefore, long-term and accurate monitoring of maize distribution is of great significance for reducing economic losses and ensuring food security^25^.

At present, methods based on remote sensing data are the basic approach for identifying the regional-scale maize planting area and its dynamics. Remote sensing data has the advantages of temporal and spatial continuity, high updating frequency, and low acquisition cost. Currently, many studies have attempted to map the distribution of maize at the provincial and national levels in China based on remote sensing data^26,27^. For example, Zhang *et al*.^26^ mapped the distribution of maize in 11 provinces in Northeast and North China in 2010 using MODIS (Moderate Resolution Imaging Spectroradiometer) data with a spatial resolution of 250 m. Luo *et al*.^27^ generated a 1000-m spatial resolution dataset of the distribution of rice, wheat, and maize in China from 2000 to 2015 based on MODIS data, by comparing the phenological periods of each pixel with the reference phenological periods of the three crops. However, China is one of the countries with the most severe cropland fragmentation issues^28^. Since the implementation of the household contract responsibility system in 1979, the absolute average allocation of land has exacerbated the degree of cropland fragmentation, resulting in extremely scattered cropland in China^29–31^. The report shows that the average cropland area per household in China was 0.58 hectares in 2014^32^. In addition, there are significant differences in planting habits among Chinese households, as farmers are free to choose which crops to plant, leading to high heterogeneity in crop types. Thus, one MODIS pixel with resolutions of 250 to 1000 m typically covers the fields of 10 to 172 households, resulting in a large amount of misclassification that affects the accuracy of maize maps^31^.

There have been several efforts to generate distribution maps of maize using high spatial resolution satellite datasets, which can effectively avoid mixed-pixel issues. You *et al*.^33^ produced annual crop maps for the main crops (maize, soybean, and rice) of Northeast China from 2017 to 2019 based on Sentinel-2 data at 10-m resolution, with an overall accuracy of 81–86%. Recently, Shen *et al*.^22^ obtained a 30-m resolution maize map for 22 provinces of China from 2016 to 2020 using Landsat and Sentinel-2 satellite data, with an average overall accuracy of 79.13%. These studies have focused more on recent years’ maize mapping. However, a long-term high-resolution dataset of maize distribution in China is still lacking. The limitation is mainly due to the lack of corresponding long-term high spatiotemporal resolution remote sensing data. Although Landsat data have a high spatial resolution (30 m) and a long-term span, their temporal resolution is low (16 days) and tends to be affected by cloud contamination. In Southern China, for example, from 1984 to 2017, there were fewer than 10 cloud-free observations per year, making it challenging to accurately map maize due to the lack of critical crop growth information^34^. Sentinel-2 data (10 m or 20 m, 5 days) can balance spatial and temporal resolution well but lack historical time series due to the more recent launch of the satellite (e.g., Sentinel-2A was launched in 2015).

A fused data product has the potential to overcome the above problems. It can fuse frequently revisited but coarse spatial resolution images and infrequently revisited but high spatial resolution images to reconstruct long-term high spatiotemporal resolution images, providing data support for improving crop classification accuracy^35–40^. For example, Yin *et al*.^41^ produced a rice distribution map of the Sanjiang Plain by fusing MODIS and Landsat data, and the results showed that the overall accuracy improved by 6.07% based on the fused data compared to using only Landsat data. Ding *et al*.^42^ used a fusion of MODIS and Landsat data to identify rice in Nanchang County of China and achieved an overall accuracy of 93.66%. However, these studies were conducted on small reference datasets and the long-term identification of maize over large regions based on fused data still lacking. Recently, a study has combined MODIS data and Landsat data to generate a long-term high spatiotemporal resolution fused dataset of Normalized Difference Vegetation Index (NDVI) (Integrating ENvironmental VarIable spatiotemporal fusion dataset, InENVI) over a large regional scale, which can be used for high-precision crop mapping^43^.

Therefore, based on the newly produced high spatiotemporal resolution and long time series (2001–2020) NDVI fused dataset (i.e., InENVI) in China, this study produced high-resolution maps of maize distribution from 2001 to 2020 by the Time-Weighted Dynamic Time Warping (TWDTW) method. The specific objectives were: (1) to generate a publicly available database of long-term high-resolution maize distribution maps in China; (2) to evaluate the accuracy of the maize distribution dataset using field surveys, Google Earth samples, unmanned aerial vehicle images (UAV), and county-level statistical data; and (3) to analyse the temporal and spatial variation characteristics of maize distribution in China. The generated dataset provides important foundational data for estimating maize production, identifying maize phenology, and monitoring food security.

## Methods

### Study area

**Fig. 1:**
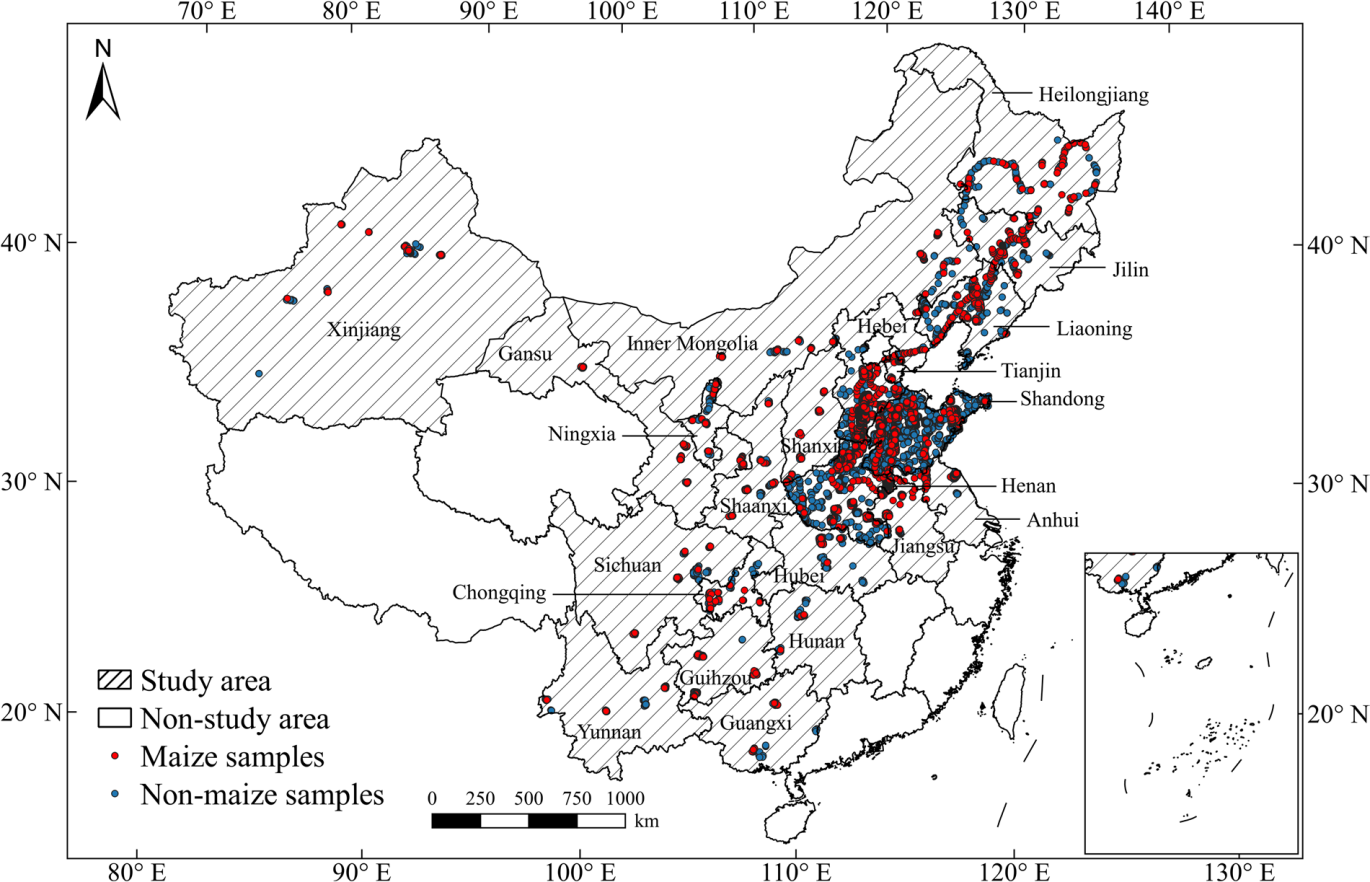
Location of study area and field survey samples. Red dots indicate maize samples and blue dots indicate non-maize samples, including other crops, forests, shrubs, water, buildings, and sheds. All samples were collected in 2019 through field surveys and © Google Earth.

**Fig. 2:**
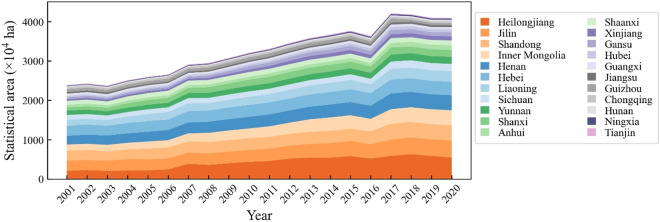
Changes in maize planting area in 22 provinces and municipalities in China from 2001 to 2020.

Based on agricultural statistical data, 30 provinces and municipalities in mainland China planted maize in 2020. This study focused on mapping maize in 22 of these provinces and municipalities, accounting for over 99% of the total maize planting area in China for 2020, including Anhui, Gansu, Hebei, Heilongjiang, Henan, Inner Mongolia, Jiangsu, Jilin, Liaoning, Ningxia, Shaanxi, Shandong, Shanxi, Tianjin, Xinjiang, Chongqing, Guangxi, Guizhou, Hubei, Hunan, Sichuan, and Yunnan (Fig. 1). In 2020, the 22 provinces produced a total of 259 billion tons of maize, with a total planting area of 40.93 million hectares (http://www.moa.gov.cn/), which increased by about 17.05 million hectares from 2001 (Fig. 2). The major maize planting provinces in China, including Heilongjiang, Jilin, Shandong, Inner Mongolia, Henan, Hebei, and Liaoning, accounted for over half of the total planting area in China.

In China, maize can be divided into spring maize and summer maize based on the sowing season. Summer maize is mainly planted in the Huang-Huai-Hai region, including Henan, Shandong, Jiangsu, and Anhui; Hebei, Shanxi, Shaanxi, and Xinjiang plant both spring maize and summer maize; other provinces mainly plant spring maize. Summer maize is usually rotated with other winter crops (such as summer maize-winter wheat rotation). The planting time is typically from late May to mid-to-late June, with harvest occurring from mid-to-late September to early October. Spring maize is only planted once a year, with a wide range of planting times across different regions, from March to May. The harvest time varies depending on the planting time, mostly from August to October.

### Fused dataset

In this study, the latest fused InENVI NDVI dataset was used to generate the maize distribution maps in China^43^. This dataset is based on the nonlinear relationship between MODIS NDVI and Landsat NDVI to reconstruct high spatial resolution NDVI data^43^. The dataset has a wide spatial coverage (China), long time series (2001–2020), and high spatiotemporal resolution (30 m, 8 days), making it a high-quality dataset for long-term identification of maize planting areas.

### Agriculture data

This study assessed the accuracy of a maize distribution map through field surveys and statistical data. In 2019, field surveys were conducted at 600 randomly selected sites across 11 provinces in China. In August 2018, three UAV (eBee, senseFly Ltd., Switzerland) images covering approximately 0.1 square kilometers and containing about 6000 spatial resolution 30 × 30 m pixel samples were collected in Ningxia, Shaanxi, and Inner Mongolia. Additionally, maize fields and other land cover types were interpreted using high-resolution satellite images from Google Earth in 22 provinces for 2002, 2005, 2007, 2008, 2011, 2012, 2013, and 2019. In total, 54,281 samples with a spatial resolution of 30 × 30 m were obtained through field surveys and visual interpretation of Google Earth. Among them, 22,070 samples were maize, and 32,211 samples were other crops, forests, shrubs, water, buildings, and sheds.

In addition, this study evaluated the accuracy of the identified maize distribution using county-level and provincial-level statistical data obtained from the National Bureau of Statistics (NBS) of China (http://data.stats.gov.cn/). Accuracy assessment was conducted using county-level statistical data from 17 provinces from 2001 to 2020.

### Time-weighted dynamic time warping

In this study, the TWDTW method proposed by Maus *et al*.^44^ and Dong *et al*.^45^ was used to identify the spatial distribution of maize in China, which run annually. The TWDTW method is an improved version of the Dynamic Time Warping (DTW) algorithm, which measures the similarity between two non-linear time series curves by calculating the distance value. Assuming that the time series **X = **{*x*_1_, *x*_2_, …, *x*_*n*_} is the curve of an unknown pixel, and the time series **Y** = {*y*_1_, *y*_2_, …, *y*_*m*_} is the standard curve of a known maize pixel, and the lengths of the two curves are n and m respectively. The DTW algorithm measures the similarity between the two given time series using the Euclidean distance and can flexibly warp and stretch the time series **X** to align with the time series **Y**. Use *d*_*base*(*i*, *j*)_ to represent the distance matrix obtained by calculating the Euclidean distance between any two points in sequence X and sequence Y. The calculation is as follows^46^:

1 $$\begin{array}{c}{d}_{base\left(i,j\right)}=\left|{x}_{i}-{y}_{j}\right|\end{array}$$

Among them, *x*_*i*_ ∈ **X** ∀ *i* = 1, 2, …, *n*, *y*_*j*_ ∈ **Y** ∀ *j* = 1, 2, …, *m*. Each matrix element *d*_(*i*, *j*)_ represents the alignment distance between *x*_*i*_ and *y*_*i*_.

On the basis of the distance matrix *d*_*base*(*i*, *j*)_, the cumulative distance matrix is obtained by recursively summing the minimum distance *d*_*i*, *j*_:

2 $$\begin{array}{c}{d}_{i,j}={d}_{base\left(i,j\right)}+min\left\{{d}_{i-1,j},{d}_{i-1,j-1},{d}_{i,j-1}\right\}\end{array}$$

The DTW algorithm calculates the distance between two sequences by finding a warping path with the smallest stretching or compressing distance in the cumulative distance matrix, and the points on the path are the points where the two sequences are warped and aligned. The final distance is used to characterize the similarity between the time series **X** and **Y**. TWDTW improves DTW by adding time weights, which avoids over-stretching or compressing the curves during time matching and ignoring the seasonal variation of crops. The calculation of the distance matrix *d*_*base*(*i*, *j*)_ in TWDTW becomes:

3 $$\begin{array}{c}{d}_{base\left(i,j\right)}={\omega }_{i,j}+\left|{x}_{i}-{y}_{j}\right|\end{array}$$

4 $$\begin{array}{c}{\omega }_{i,j}=\frac{1}{1+{e}^{-\alpha \left(g\left({t}_{i},{t}_{j}\right)-\beta \right)}}\end{array}$$

This study calculated the time weights using a logistic model with parameters suggested by Belgiu and Csillik (2018), where the steepness and midpoint were set to 0.1 and 50, respectively, indicating lower penalties for time warping less than 50 days and higher penalties for time warping more than 50 days.

**Fig. 3:**
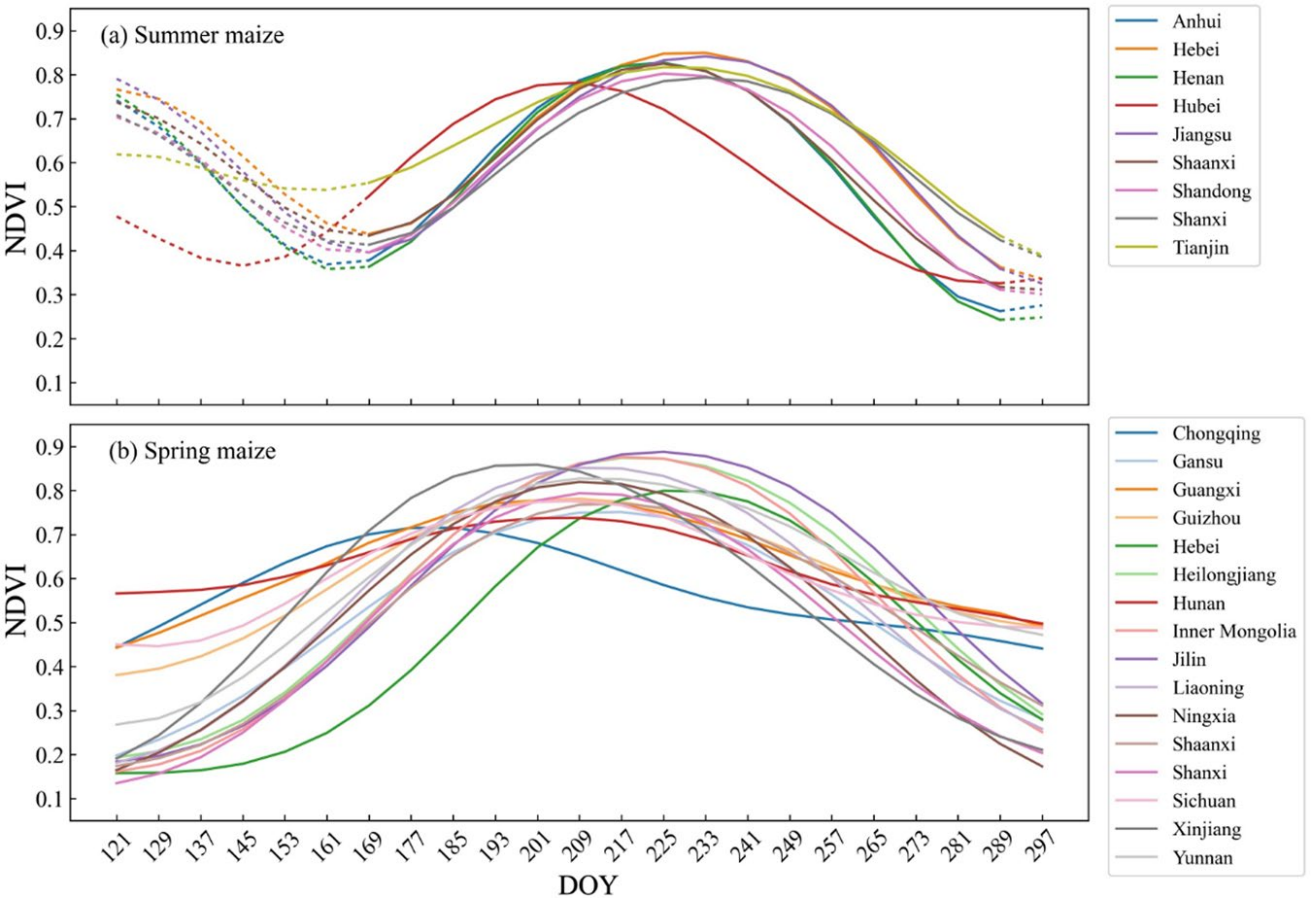
Standard seasonal curves of maize in 22 provinces in 2019, including (**a**) summer maize and (**b**) spring maize.

First, this study created a potential maize distribution map based on the NDVI time series, where pixels with NDVI greater than 0.3 at any time during the maize growing period to reduce the number of identified pixels. Then, fifty field samples of maize were randomly selected in each province and their NDVI time series were averaged to obtain the standard seasonal curve of NDVI for spring and summer maize (Fig. 3). The similarity between the standard NDVI seasonal curve and the seasonal curve for the unknown land cover type was calculated for each pixel, with higher similarity indicating a higher probability of the identified pixels as maize. For the provinces planted both spring and summer maize, we calculated the similarity between the seasonal curve and the two standard curves for each pixel separately, and took the one with the higher similarity as the correct similarity for the pixel. Finally, we selected the n pixels with the highest similarity to the standard curve as the identified maize pixels. The total area of all selected n pixels should be equal to the planting area of maize in the given province. The similarity threshold for each province was determined using the provincial statistical area of maize.

### Statistical analysis

In this study, the identification accuracy of maize was evaluated based on the field surveys conducted in 2019. Fifty field samples were randomly selected to determine the standard seasonal curve of maize. The remaining samples were used to calculate three accuracy indicators, including Producer’s Accuracy (PA), User’s Accuracy (UA), and Overall Accuracy (OA). PA represents the percentage of the surveyed maize samples correctly identified as maize; UA represents the percentage of identified maize that are actually confirmed as maize samples by field survey; OA is calculated as the percentage of correctly identified samples. The three accuracy metrics can be calculated as:

5 $$\begin{array}{c}{\rm{PA}}=\frac{TP}{TP+FP}\times 100 \% \end{array}$$

6 $$\begin{array}{c}{\rm{UA}}=\frac{TP}{TP+FN}\times 100 \% \end{array}$$

7 $$\begin{array}{c}{\rm{OA}}=\frac{TP+TN}{TP+TN+FP+FN}\times 100 \% \end{array}$$

where *TP* is the number of correctly classified maize samples, *TN* is the number of correctly classified non-maize samples, *FP* is the number of non-maize samples classified as maize, and *FN* is the number of maize samples classified as non-maize.

In addition, this study compared the identified maize planting area with the county-level statistical area. The coefficient of determination (*R*^2^), the slope of the regression line between the identified area and the statistical area, and the relative mean absolute error (RMAE) were calculated for the 17 provinces with county-level statistical data. The calculation formulas of *R*^2^ and RMAE are as follows:

8 $${R}^{2}=1-\frac{{\sum }_{i=1}^{n}{\left(I{A}_{i}-S{A}_{i}\right)}^{2}}{{\sum }_{i=1}^{n}{\left(\overline{SA}-S{A}_{i}\right)}^{2}}$$

9 $$RMAE=\frac{{\sum }_{i=1}^{n}\left|S{A}_{i}-I{A}_{i}\right|}{{\sum }_{i=1}^{n}S{A}_{i}}$$

where *SA*_*i*_ and *IA*_*i*_ are the statistical area and identified area of the *i*th county, and n represents the number of counties in a given province.

## Data Records

The 30 m CCD-Maize dataset from 2001 to 2020 is available at 10.57760/sciencedb.08490^47^. The dataset is provided in GeoTIFF format, with pixel values of 1 for maize and 0 for non-maize. A total of 440 GeoTIFF files are stored under 20 folders, and each folder represents the maize maps of 22 provinces in a specific year from 2001 to 2020.

## Technical Validation

### Accuracy assessment

#### Field survey assessment

**Fig. 4:**
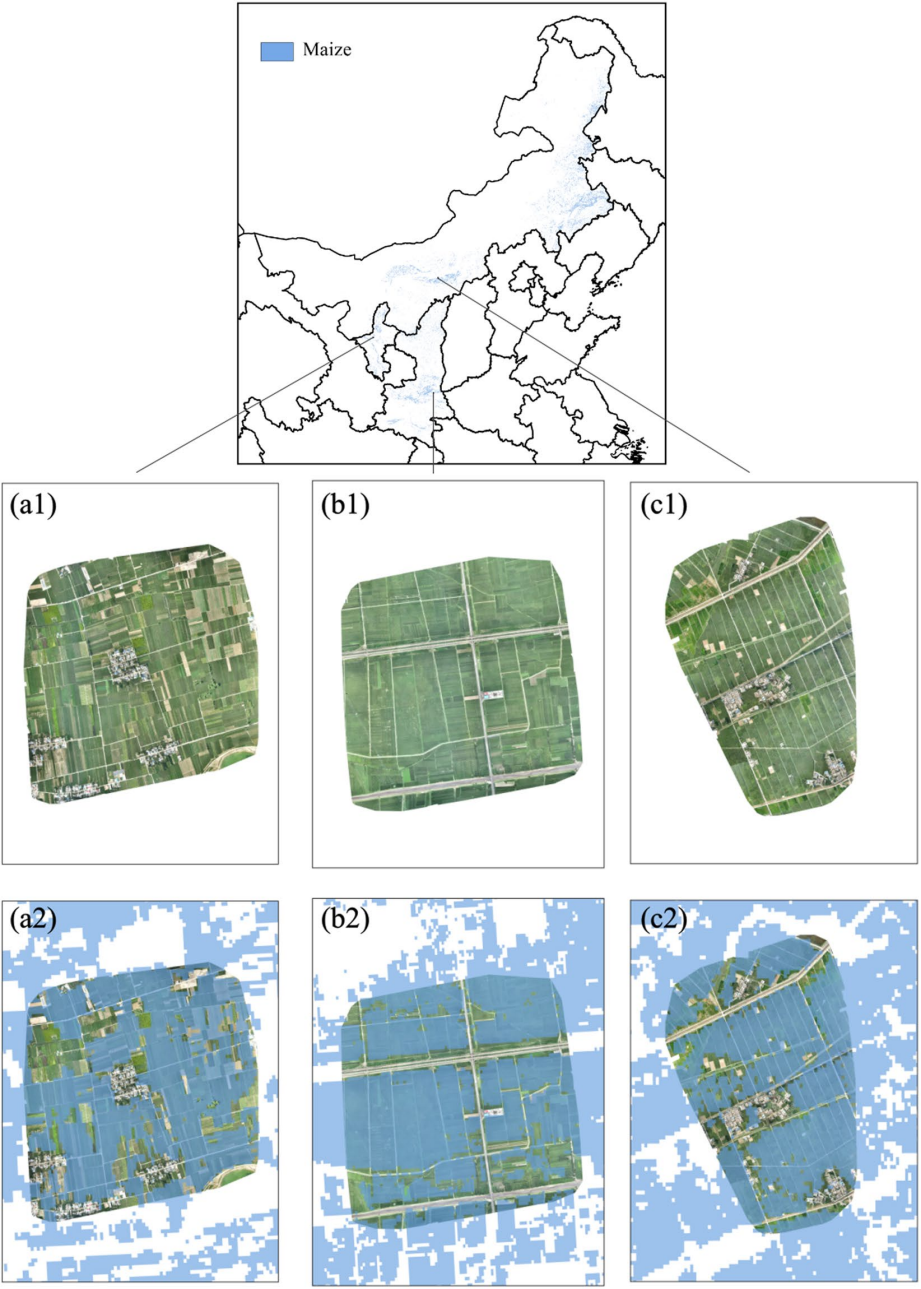
Classification maps at UAV sites of Ningxia (a1,a2), Shaanxi (b1,b2), and Inner Mongolia (c1,c2). All UAV pictures were taken in August 2018. Red indicates the identified maize pixel. The coordinates of the center points of a1 and a2 are (105°48′36″ E, 37°32′24″ N); the coordinates of the center point of b1 and b2 are (109°44′04″ E, 34°33′0″ N); the coordinates of the center point of c1 and c2 are (110°27′0″ E, 40°33′13″ N).

We quantitatively evaluated the accuracy of maize distribution maps based on field survey samples from 2002, 2005, 2007, 2008, 2011, 2012, 2013, and 2019. On average, the OA of maize identification in 22 provinces was 80.06%, with UA and PA of 77.32% and 80.98%, respectively (Tables 1, 2). Jilin had the highest OA of 94.04%, while Chongqing had the lowest at 64.81% (Tables 1, 2). There was a large variation in UA and PA across provinces, with lower accuracy found in several provinces in Southern China, such as Hunan, Hubei, Jiangsu, Guangxi, Chongqing, and Guizhou (Table 2). In some provinces, such as Henan Province, the accuracy varies greatly between early years and recent years, with the OA of 82.72% in 2019 and only 53.06% in 2013 (Table S1). Figure 4a1,b1,c1 show large maize fields captured by UAV in August 2018 at three sites in Ningxia, Shaanxi, and Inner Mongolia, with most fields showing a dark green color indicating maize, and a small area of other crops (such as rice, displaying light green). As shown in Fig. 4, the maize distribution map produced in this study accurately identified the location of maize and distinguished buildings and wide roads. However, there were some misclassifications, with some light green rice fields being misclassified as maize (Fig. 4a2).

**Table 1:** Confusion matrices of the distribution map of maize in northern provinces.

Province	Class	Maize	Other	UA (%)	PA (%)	OA (%)
Gansu	Maize	190	82	82.97	69.85	79.46
	Other	39	278	77.22	87.70	
Hebei	Maize	2137	156	83.77	93.20	89.16
	Other	414	2549	94.23	86.03	
Heilongjiang	Maize	921	130	56.50	87.63	83.26
	Other	709	3251	96.15	82.10	
Henan	Maize	1145	544	55.83	67.79	76.43
	Other	906	3556	86.73	79.70	
Inner Mongolia	Maize	1756	237	72.38	88.11	79.75
	Other	670	1816	88.46	73.05	
Jilin	Maize	811	36	84.48	95.75	94.04
	Other	149	2106	98.32	93.39	
Liaoning	Maize	1906	28	82.98	98.55	91.57
	Other	391	2647	98.95	87.13	
Ningxia	Maize	592	498	89.97	54.31	71.67
	Other	66	835	62.64	92.67	
Shaanxi	Maize	327	95	89.10	77.49	89.00
	Other	40	765	88.95	95.03	
Shandong	Maize	4837	504	83.20	90.56	88.58
	Other	977	6645	92.95	87.18	
Shanxi	Maize	485	175	83.62	73.48	87.27
	Other	95	1366	88.64	93.50	
Tianjin	Maize	74	34	88.10	68.52	76.72
	Other	10	71	67.62	87.65	
Xinjiang	Maize	69	21	54.33	76.67	75.16
	Other	58	170	89.01	74.56	

The rows in the confusion matrices mean the number of field identified samples, and the columns mean the number of surveyed samples.

**Table 2:** Confusion matrices of the distribution map of maize in southern provinces.

Province	Class	Maize	Other	UA (%)	PA (%)	OA (%)
Anhui	Maize	603	96	68.37	86.27	78.06
	Other	279	731	88.39	72.38	
Chongqing	Maize	12	0	24.00	100.00	64.81
	Other	38	58	100.00	60.42	
Guangxi	Maize	13	6	26.00	68.42	66.41
	Other	37	72	92.31	66.06	
Guizhou	Maize	34	25	64.15	57.63	73.65
	Other	19	89	78.07	82.41	
Hubei	Maize	158	99	45.80	61.48	80.97
	Other	187	1059	91.45	84.99	
Hunan	Maize	9	0	27.27	100.00	76.24
	Other	24	68	100.00	73.91	
Jiangsu	Maize	168	41	82.35	80.38	81.88
	Other	36	180	81.45	83.33	
Sichuan	Maize	76	30	60.80	71.70	77.87
	Other	49	202	87.07	80.48	
Yunnan	Maize	270	9	48.74	96.77	79.28
	Other	284	851	98.95	74.98	

The rows in the confusion matrices mean the number of field identified samples, and the columns mean the number of surveyed samples.

#### Statistical data assessment

**Fig. 5:**
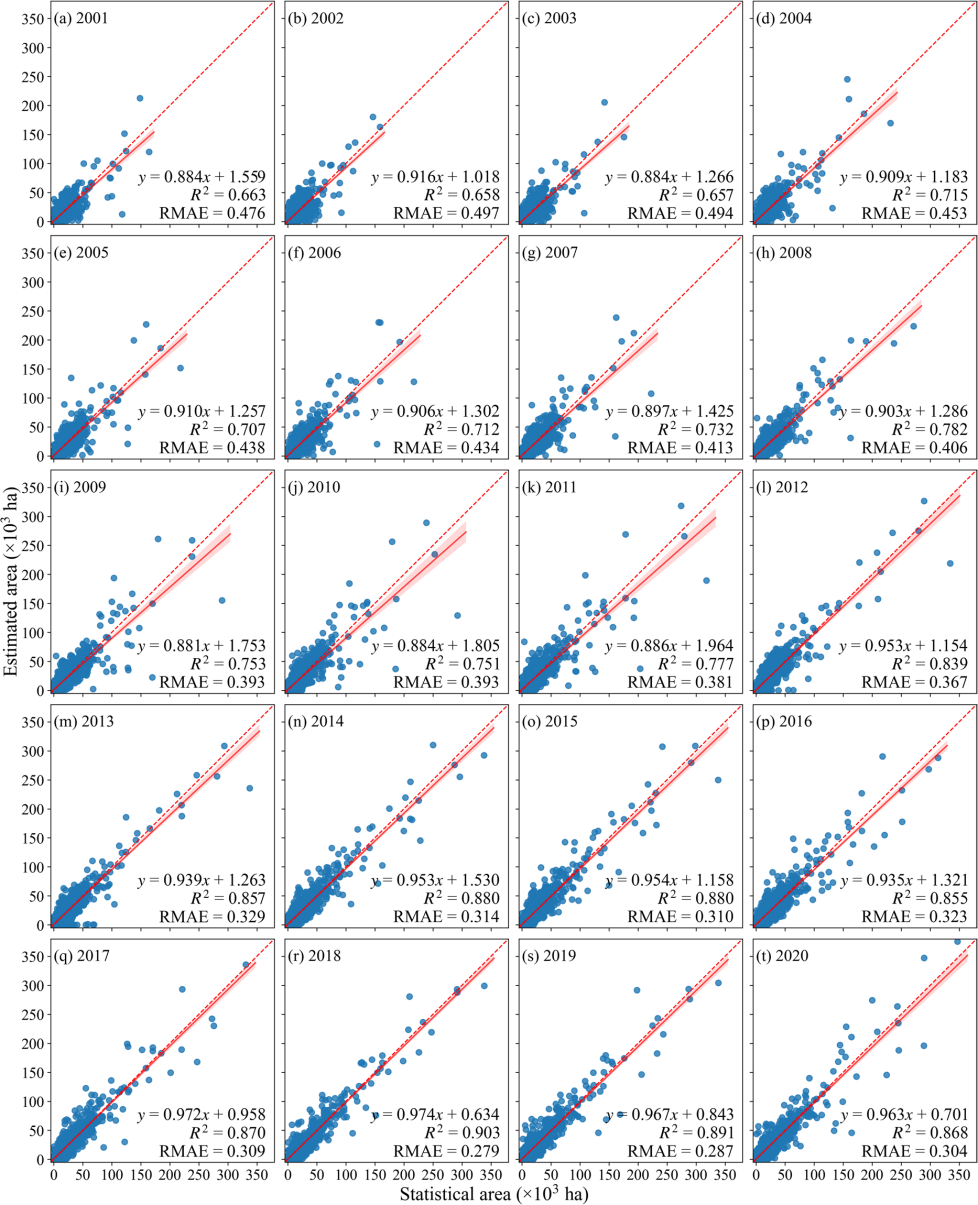
County-level comparison of identified and statistical planting areas of all provinces. (**a**–**t**) show 2001–2020. The red dashed lines indicate the 1:1 line, and the red solid lines indicate the regression lines.

The accuracy of the maize maps was further validated based on county-level statistical data in this study. A total of 17 provinces with county-level statistical data were used for validation, with the total number of counties ranging from 1131 to 1614. The results showed that the maize distribution maps produced in this study could effectively reproduce the spatial variability of the maize planting area (Fig. 5). From 2001 to 2020, the identified areas and the statistical areas of maize showed a strong correlation, with the scatters concentrated near the 1:1 line. The slope of the regression line between the maize classified area and the statistical area ranged from 0.881 to 0.974, the RMAE ranged from 0.279 to 0.497, and the *R*^2^ was above 0.65, with a maximum of 0.903 in 2018 (Fig. 5).

**Fig. 6:**
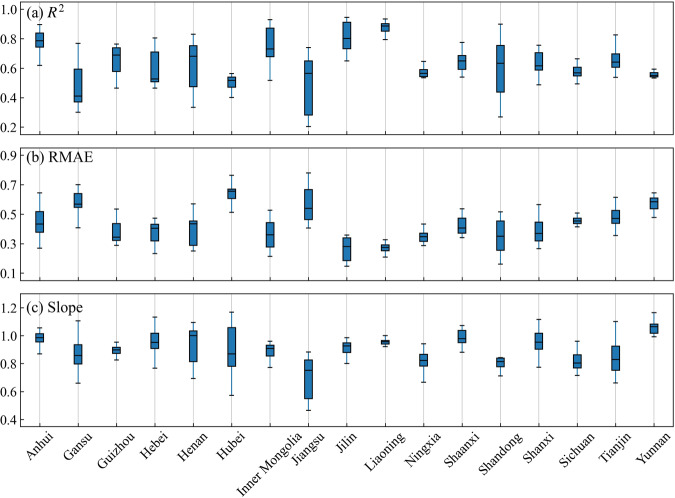
County-level comparison of identified and statistical planting areas in each province. (**a**) *R*^2^, (**b**) RMAE, and (**c**) Slope of county-level identified maize areas compared to agricultural statistical data in 17 provinces for 2001–2020.

**Fig. 7:**
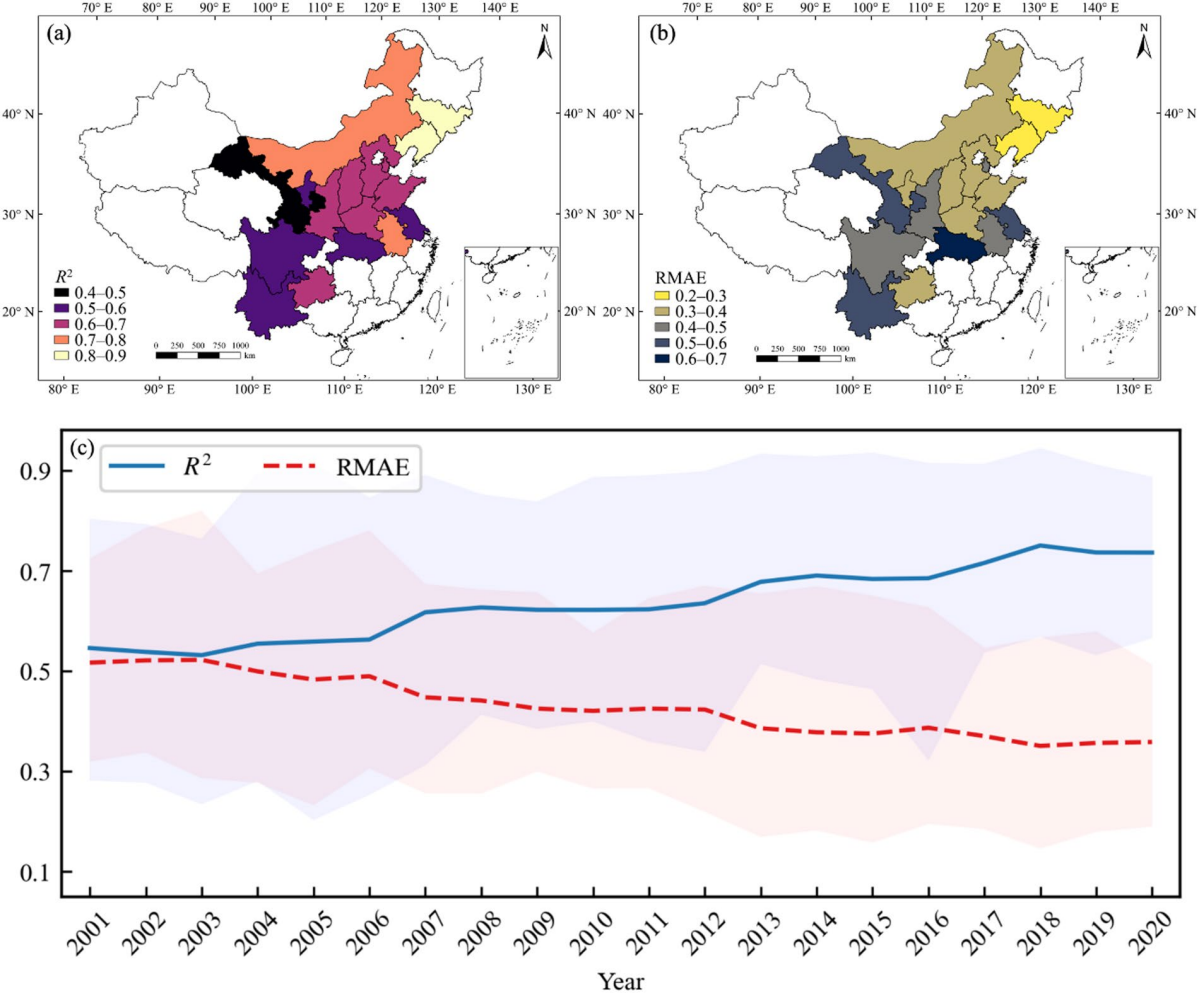
Spatial comparison of (**a**) *R*^2^ and (**b**) RMAE of identified and statistical planting areas, and (**c**) trend change in average *R*^2^ and RMAE from 2001 to 2020 over the 17 provinces with the county-level statistical area.

Moreover, this study also analyzed the consistency between the identified maize area and the statistical area for each province. The results showed that the average *R*^2^ of all provinces from 2001 to 2020 ranged from 0.477 to 0.868, the slopes of the regression line ranging from 0.712 to 1.001, and RMAE ranging from 0.264 to 0.644 (Fig. 6). As shown in Fig. 7a,b, large identification errors were found in the middle and lower reaches of the Yangtze River Plain (i.e., Jiangsu, Hubei) and mountainous regions (i.e., Gansu, Ningxia, Yunnan, Sichuan), with *R*^2^ below 0.6. The northeast region (i.e., Liaoning, Jilin) showed the best identification accuracy, with Liaoning achieving the highest *R*^2^ of 0.868, followed by Jilin with *R*^2^ of 0.813 (Figs. 6, 7a). The identification accuracy of major maize-producing provinces such as Jilin, Liaoning, and Inner Mongolia was higher than that of other provinces from 2001 to 2020. The identification accuracy of provinces such as Jiangsu, Shandong, Henan, and Gansu fluctuated greatly between 2001 and 2020. For example, the *R*^2^ of Jiangsu was only 0.203 in 2003 but reached 0.739 in 2019 (Fig. 6). From the interannual variation of the average identification accuracy, both *R*^2^ and RMAE showed significant temporal trends, with *R*^2^ gradually increasing and RMAE gradually decreasing from 2001 to 2020 (Fig. 7c). In 2018, RMAE reached a minimum of 0.350, and *R*^2^ was 0.750. Overall, the maize distribution maps produced in this study accurately reproduced the spatial variability of maize planting area and also achieved good performance at the annual scale.

### Spatiotemporal patterns of maize

**Fig. 8:**
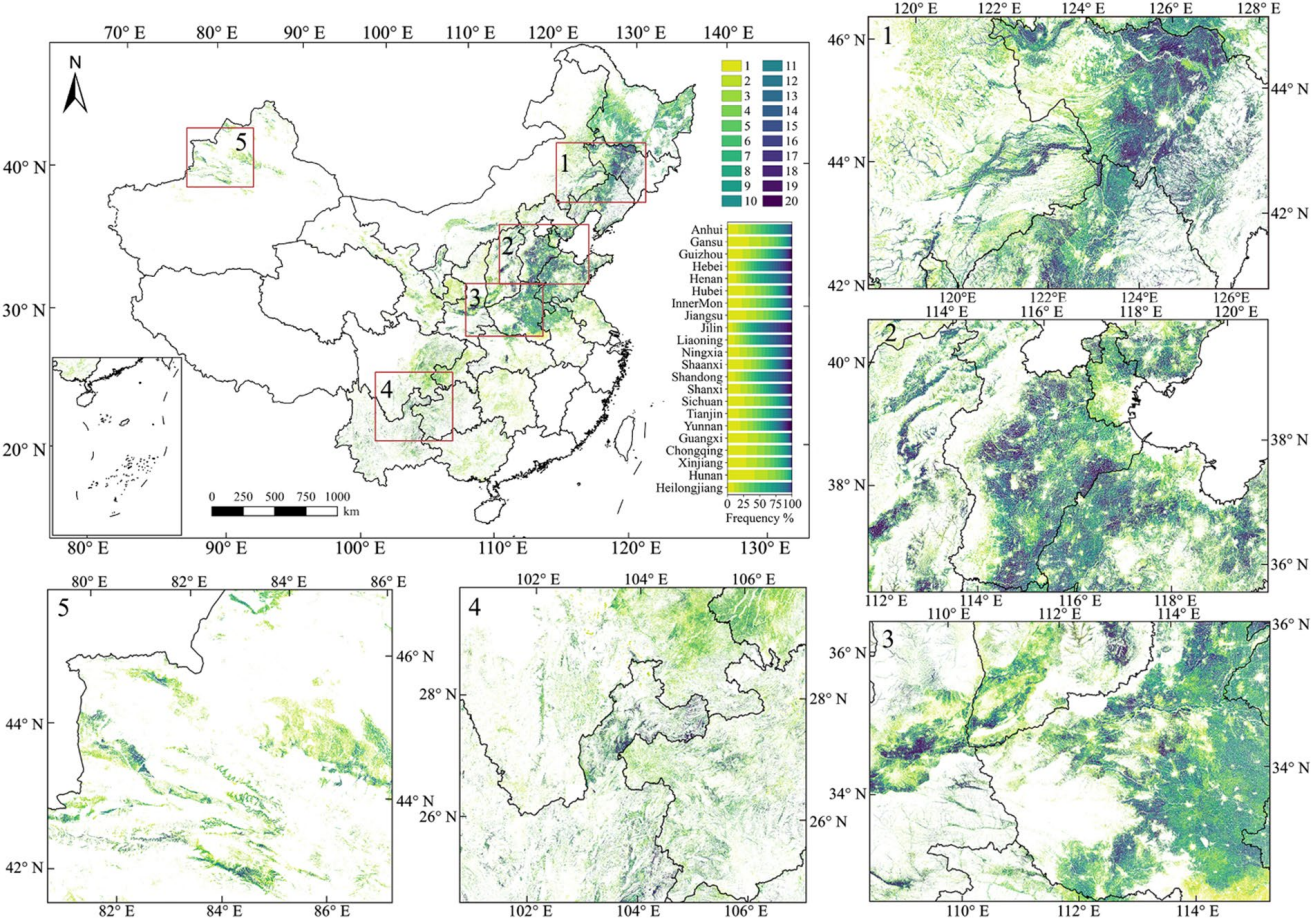
Distribution of maize planting frequency in China from 2001 to 2020. Panels 1–5 on the right and bottom are the zoomed-in maps, indicating the local details of different provinces and regions, including Northeast, North China, South China, Southwest, and Xinjiang.

Based on the maize distribution maps generated by this study, we first analyzed the ratio of pixels with continuous maize planting in China from 2001 to 2020. As shown in Fig. 8, the maize planting in China generally showed a high frequency of continuous planting, with 50.74% of pixels having continuously planted maize for over 10 years and 19.80% for less than 5 years. The frequency of continuous maize plantings varied largely across different regions. The Northeast and Northern China, two major maize production regions, had relatively high frequencies of continuous maize planting, with over 67.13% of pixels planted for more than 10 years (Fig. 8). In contrast, Hunan had the lowest frequency of continuous maize planting, with 56.12% of pixels planted for less than 5 years (Fig. 8).

**Fig. 9:**
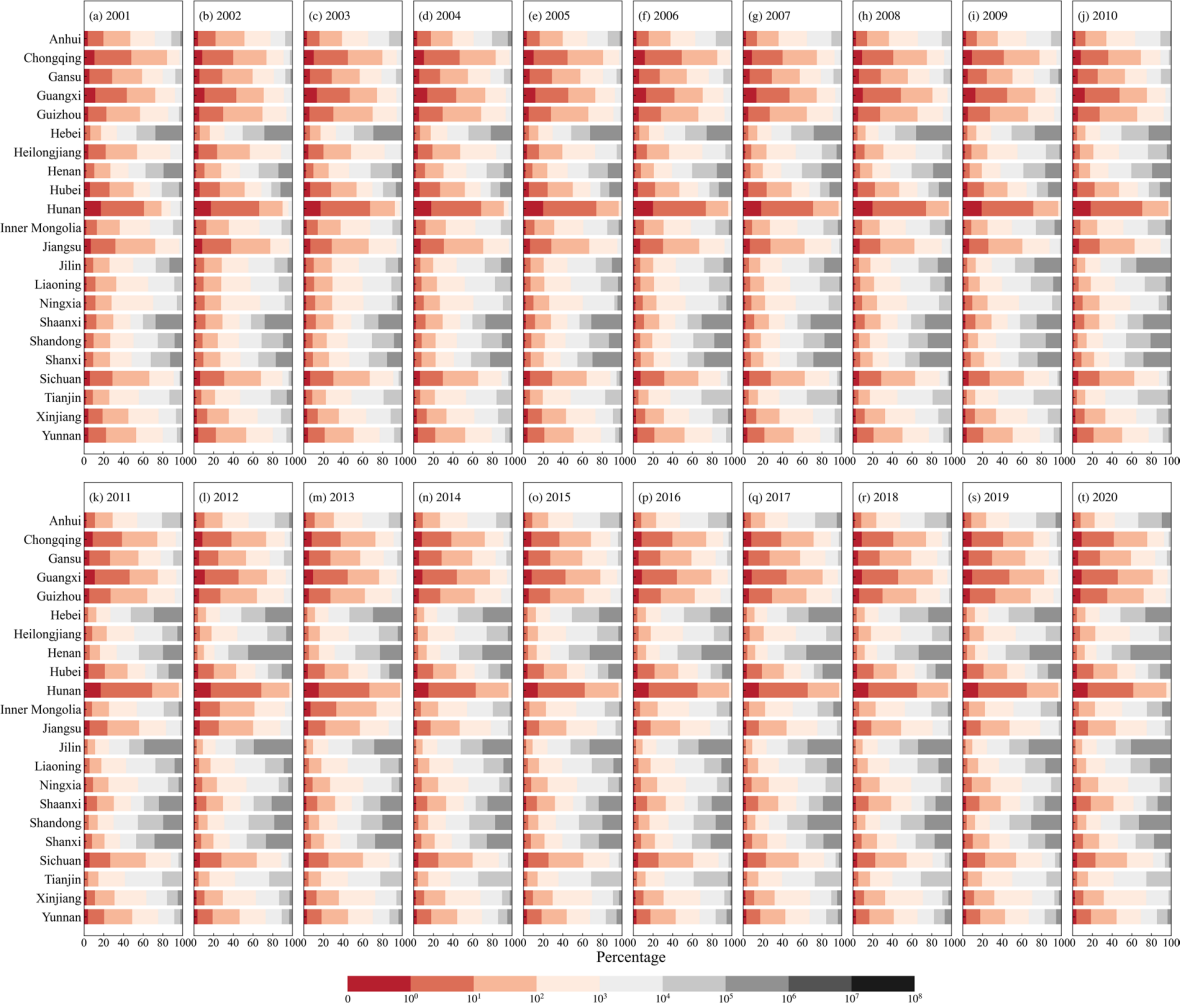
Proportion of patches with different numbers of pixels (0–10^8^) on the maize map of each province, (**a**–**t**) indicates 2001–2020.

In addition, we analyzed the distribution of different-sized patches based on the generated maize distribution maps in China (Fig. 9). As shown in Fig. 9, the proportion of large patches (defined in this study as patches with over 1,000 pixels, an area of about 90 hectares or larger) in major maize-producing provinces such as Hebei, Henan, Jilin, Liaoning, and Shandong were very high, reaching over 50%. However, in most southern provinces, such as Guangxi, Guizhou, Hubei, Hunan, Sichuan, Yunnan, and Chongqing, the proportion of large patches was relatively low, at only 13.88%. The proportion of patches of different sizes in each province depends largely on the degree of cropland fragmentation in China. In this study, we used the proportion of small patches (defined in this study as patches with 10 or fewer pixels, an area of about 0.9 hectares or less) as an indicator to measure the fragmentation of maize distribution maps. From 2001 to 2020, the most severely fragmented areas were generally in South and Southwest China, while the degree of fragmentation in Northeast China was much smaller (Fig. 9).

**Fig. 10:**
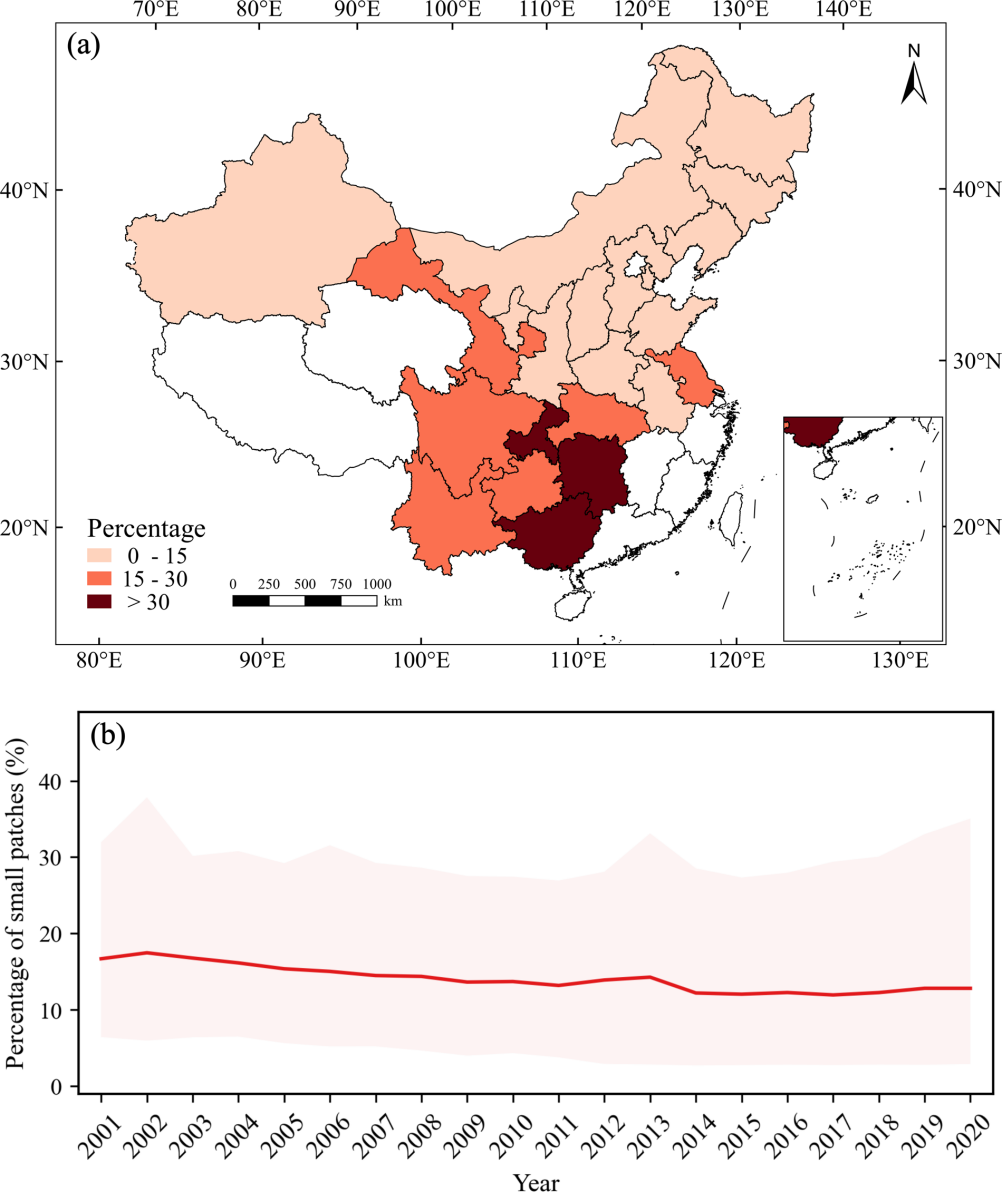
Statistics of the proportion of patches with 10 or fewer pixels (small patches). (**a**) spatial distribution of the proportion of small patches, and (**b**) the distribution of the average proportion of small patches in all provinces from 2001 to 2020.

The fragmentation of maize maps can be further classified into three classes (Fig. 10a). Class I includes provinces with a small proportion of small patches (less than 15%), including Heilongjiang, Inner Mongolia, Jilin, Liaoning, Henan, Hebei, Shanxi, and Shandong, which are major maize planting provinces. Class II has a proportion of small patches between 15% and 30%, including Yunnan, Guizhou, Sichuan, Jiangsu, Hubei, and Gansu. Class III has a proportion of small patches greater than 30%, including Chongqing, Guangxi, and Hunan. Among them, Hunan has the highest proportion of small patches, reaching 67.52%, indicating that maize planting in this province is highly fragmented. Provinces with higher proportions of small patches (i.e., classes II and III) are mostly mountainous areas, where maize planting is more scattered. It should be noted that although Jiangsu is adjacent to Anhui, there is a significant difference in the proportions of small patches. This is because Jiangsu promotes rice planting to the north of the Huaihe River, where rice is widely planted, while maize planting is relatively less concentrated and more scattered, leading to a higher proportion of small patches (i.e., 24.33%). In contrast, in Anhui, north of the Huaihe River, maize is the main crop, while south of the Huaihe River, rice is the main crop, resulting in more concentrated maize planting and a lower proportion of small patches (i.e., 12.80%) (Fig. 10a). Additionally, the average proportion of small patches has gradually decreased from 2001 to 2020 (Fig. 10b), indicating a gradual reduction of cropland fragmentation in China.

### Comparison with other studies

**Fig. 11:**
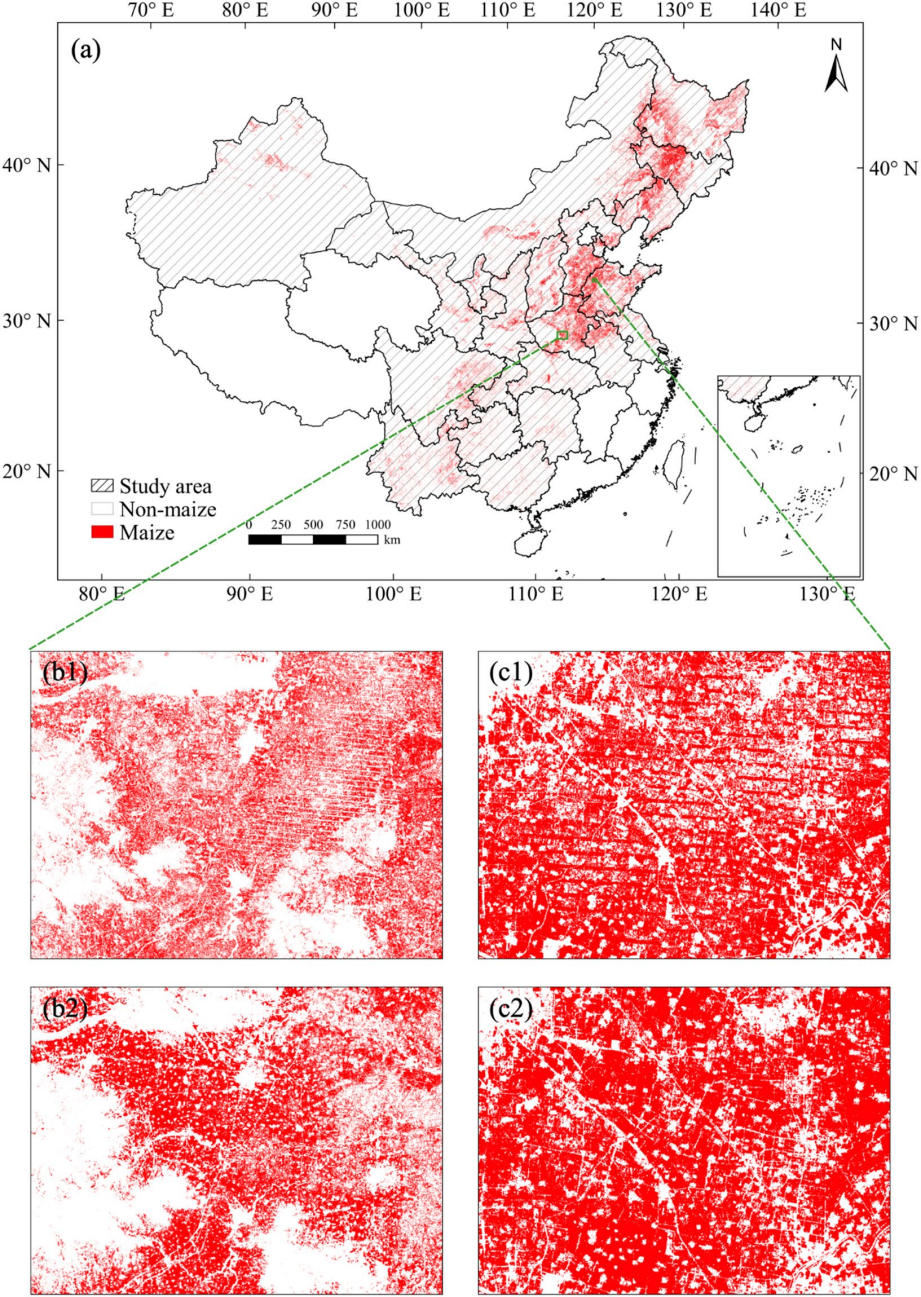
Comparison of the maize map of this study and Shen *et al*.^22^. (**a**) Maize planting area mapping based on fused products of 2020 in China; (b1,c1) partial zoomed-in maps of the identification results of Shen *et al*.^22^; (b2,c2) partial zoomed-in maps of the identification results based on the fused dataset.

We compared the dataset produced in this study with an existing product, a 30-m maize identification product for China from 2017 to 2020, produced by Shen *et al*.^22^, which was based on the NDVI composited from Landsat 7, Landsat 8 and Sentinel-2 images. Taking 2020 as an example, we selected study areas in Henan and Shandong (see the green boxes in Fig. 11a) and compared two products. As shown in Fig. 11b1,c1, the maize identification map of Shen *et al*.^22^ showed clear striping problems. This is due to the large number of missing values in these areas of Landsat and Sentinel-2 data, which cannot be fully restored by common filling interpolation methods^48^, thus affecting the identification accuracy^45^. Instead, the maize distribution map produced in this study, which was based on the NDVI fused dataset, overcame this problem well, and the identified maize fields are more complete (Fig. 11b2,c2).

**Fig. 12:**
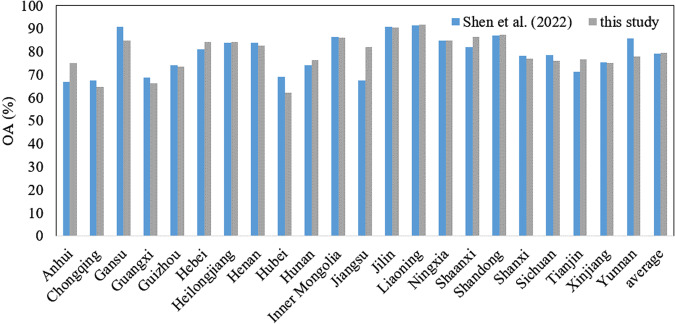
Comparison of the overall accuracy of the maize planting distribution map in 2019 of this study and Shen *et al*.^22^.

Based on field survey data of 2019, the average OA of this study is slightly higher than that of Shen *et al*.^22^. Specifically, the OA in Anhui and Jiangsu have significantly improved compared to Shen *et al*.^22^, while those in Gansu and Yunnan provinces have decreased (Fig. 12). In addition, this study calculated the average *R*^2^ at the county level from 2017 to 2020, and the results show that the average *R*^2^ based on the fused product was 0.877, which is higher than the identification accuracy of Shen *et al*.^22^, which was 0.822. Overall, the maize distribution maps produced in this study have achieved higher identification accuracy than the product of Shen *et al*.^22^.

**Fig. 13:**
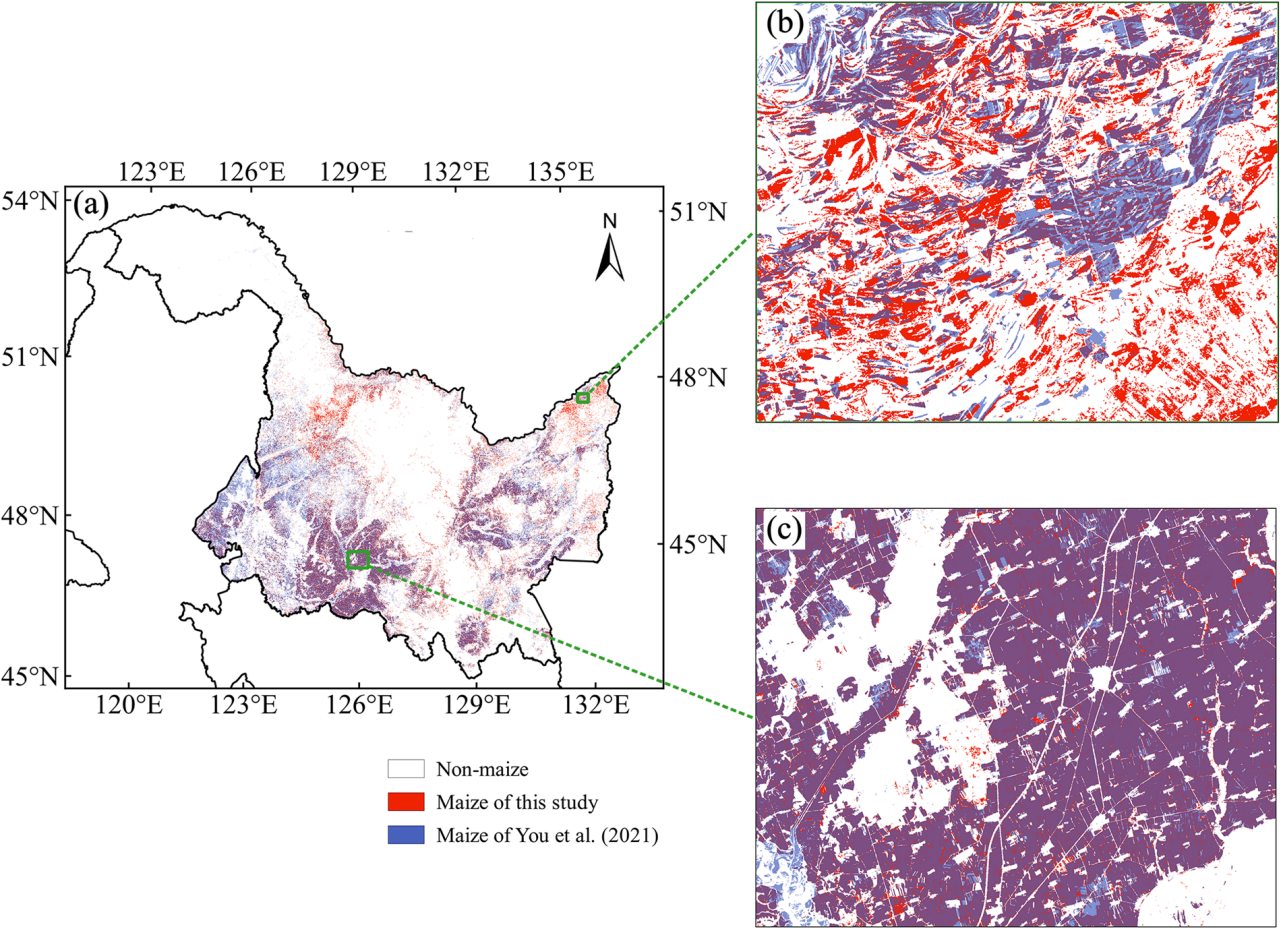
Comparison of the maize map in Heilongjiang for 2019 of this study and You *et al*.^33^. (**a**) Maize planting area mapping based on this study and You *et al*.^33^ (**b,c**) partial zoomed-in maps of this study and You *et al*.^33^.

Additionally, we further used independent sample sets from You *et al*.^33^ to verify the identification accuracy of our maize maps. On average, the OA of maize identification in 3 provinces was 85.85%, with UA and PA of 80.95% and 82.80%, respectively (Table 3). Liaoning got the highest OA of 91.50%, while Heilongjiang achieved the lowest OA of 78.52% (Table 3). In addition, we compared our maize distribution map with You *et al*.’s^33^, and the overlap percentage in Heilongjiang, Jilin, and Liaoning are 72.37%, 77.56%, and 78.08%, respectively. Figure 13 shows the overlap percentage of two products in Heilongjiang in 2019. Our product was overestimated in high latitude areas, and the two products achieved high consistency in lower latitude areas.

**Table 3:** Confusion matrices of the distribution map of maize in Northeast provinces using independent sample sets from You *et al*.^33^.

Province	Class	Maize	Other	UA (%)	PA (%)	OA (%)
Heilongjiang	Maize	1018	520	44.40	66.19	78.52
	Other	1275	5544	91.42	81.30	
Jilin	Maize	364	126	85.85	74.29	87.52
	Other	60	940	88.18	94.00	
Liaoning	Maize	463	60	79.83	88.53	91.50
	Other	117	1443	96.01	92.50	

The rows in the confusion matrices mean the number of field identified samples, and the columns mean the number of surveyed samples.

### Limitations and prospects

**Fig. 14:**
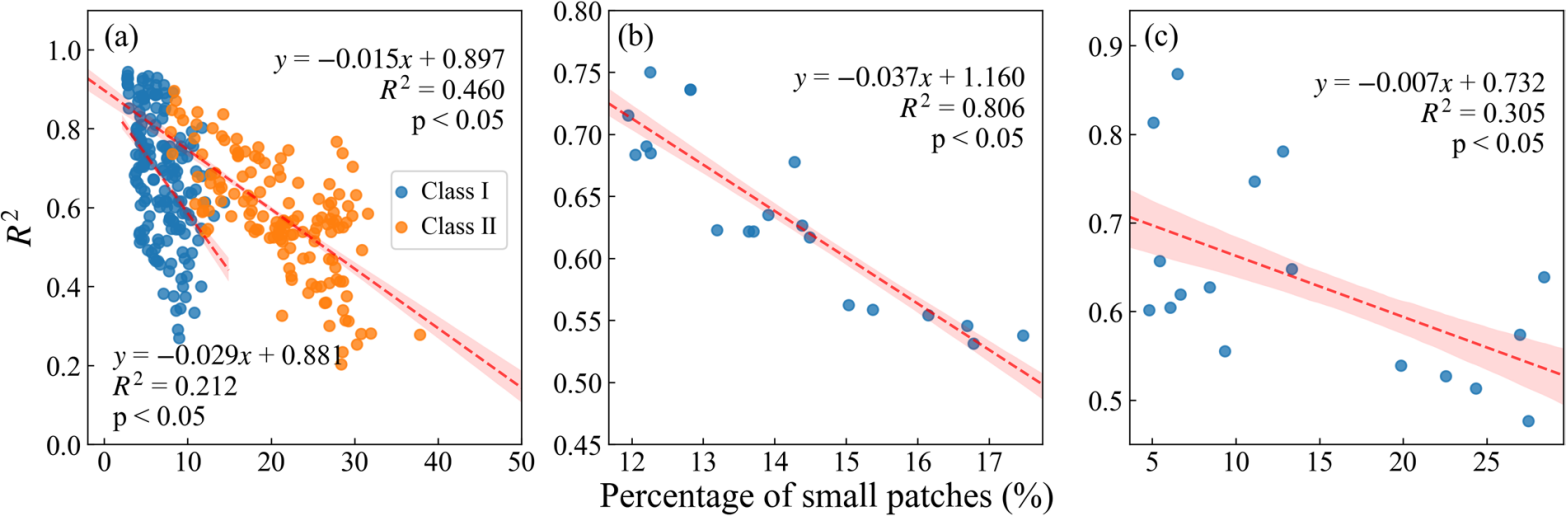
Comparison between the proportion of small patches and identification accuracy. (**a**) relationship between the proportion of the first and second types of small patches and the identification accuracy, (**b**) interannual relationship between the proportion of small patches and the identification accuracy the relationship between the changes, and (**c**) spatial comparison of the proportion of small patches and the identification accuracy.

Although the long-term annual maize distribution map produced in this study performed well in spatial and interannual variability, there are still some uncertainties. In this study, the small patch proportion was used as an indicator of cropland fragmentation to analyze the relationship between cropland fragmentation and maize identification accuracy. As shown in Fig. 14a, there was a negative correlation between maize identification accuracy and the proportion of small patches, with decreased identification accuracy as the proportion of small patches increased. When the proportion of small patches was higher (greater than 15%, i.e., Class II), the identification accuracy and the proportion of small patches showed a stronger correlation, i.e., the *R*^2^ of the Class II is 0.460, which is twice than that of Class I (0.212). In addition, the interannual variation of small patch proportion and identification accuracy shows a significant negative correlation, with an *R*^2^ of 0.806 and a slope of −0.037 (Fig. 14b). In terms of spatial distribution, they also show a certain negative correlation, with an *R*^2^ of 0.305 and a slope of −0.007 (Fig. 14c). Vintrou *et al*.^49^ also found that the identification accuracy was linearly correlated with the average patch size calculated on the crop maps (*R*^2^ = 0.8), and the identification accuracy continued to improve as the average patch size increased. Numerous studies have consistently shown that a higher degree of cropland fragmentation leads to increased uncertainty in the accuracy of crop mapping^50,51^. Overall, our results indicated that the maize identification accuracy decreased as the degree of cropland fragmentation increased. Therefore, it would be meaningless to identify the data in the other provinces and municipalities with limited and fragmentized maize areas.

**Fig. 15:**
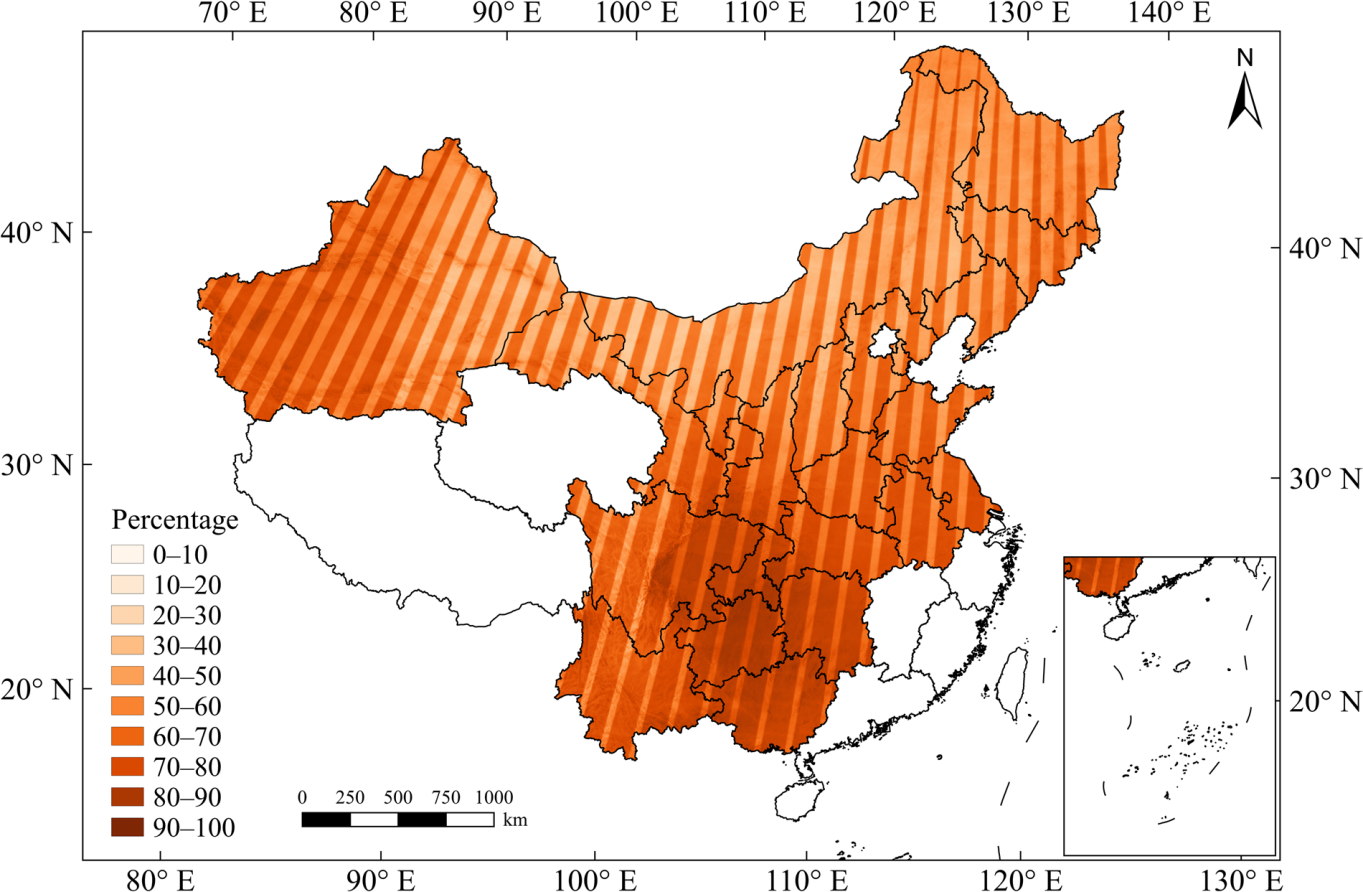
Averaged filling percentage of fused data in the study area from 2001 to 2020.

The identification accuracy is also affected by the quality of remote sensing data. A recent study found that the number of cloud-free satellite images largely determines the recovery of vegetation index seasonal changes, which in turn affects the accuracy of crop classification^45^. Although the long-term, high spatiotemporal resolution NDVI fused dataset used in this study has effectively recovered most of the missing data in the original Landsat data, the data recovery effect is still limited in some areas with severe cloud cover^52^. We calculated the proportion of Landsat missing values filled in the fused dataset in the study area from 2001 to 2020. As shown in Fig. 15, the filling percentage in Southern China was relatively high. Due to the influence of the East Asian summer monsoon in this region, there may be long periods of cloudy and rainy weather during the maize growing season, making it difficult to obtain cloud-free satellite images^53^. Taking Yunnan as an example, the average filling percentage from 2001 to 2020 was 75.26% (Fig. 15). When the filling percentage of the fused dataset is too high, it can cause some spectral distortion and blur^52^, which is an important factor to the low identification accuracy in these provinces.

The uncertainty of CCD-Maize is also highly related to the identification method used in this study, which is a phenology-based identification method. Maize has a very similar phenological cycle to many other summer crops, such as soybean, peanut, and rice, which greatly increases the difficulty of identifying maize. In most southern provinces, rice is the dominant summer crop and maize identification accuracy tends to be lower than in northern provinces (Fig. 7a,b). A previous study also found that maize had the lowest identification accuracy compared to rice and winter wheat distribution maps identified using the 250-m MODIS data^27^. Using only one index (such as NDVI) is not enough to fully distinguish maize from other summer crops^22^. To improve identification accuracy, further research is needed to explore the differences in vegetation indexes or surface albedo between maize and other summer crops. The red-edge bands from Sentinel-2 data are considered to be very useful for vegetation monitoring and are widely used for crop classification^54–56^. Currently, multi-source data fusion mainly focuses on Landsat and MODIS data. In the future, using additional satellite data for spatiotemporal fusion, such as Sentinel-2, may improve fusion accuracy, leading to higher precision maize distribution maps.

### Supplementary information

Supplementary information

## Data Availability

The classification of maize for each province in this study was performed on the local computer. The codes used is written in Python, Fortran, and Julia which are available from https://github.com/Pengqy97/TWDTW_codes.

## References

[1] FAO. *World Food and Agriculture – Statistical Yearbook 2021*. 10.4060/cb4477en (FAO, 2021).

[2] Tilman D, Balzer C, Hill J, Befort BL (2011). Global food demand and the sustainable intensification of agriculture. Proceedings of the National Academy of Sciences.

[3] Asseng S (2017). Hot spots of wheat yield decline with rising temperatures. Global Change Biology.

[4] Hochman Z, Gobbett DL, Horan H (2017). Climate trends account for stalled wheat yields in Australia since 1990. Global Change Biology.

[5] Ranum P, Peña-Rosas JP, Garcia-Casal MN (2014). Global maize production, utilization, and consumption. Annals of the New York Academy of Sciences.

[6] Dabija A, Ciocan ME, Chetrariu A, Codină GG (2021). Maize and Sorghum as Raw Materials for Brewing, a Review. Applied Sciences.

[7] Vintrou E, Ienco D, Bégué A, Teisseire M (2013). Data Mining, A Promising Tool for Large-Area Cropland Mapping. IEEE Journal of Selected Topics in Applied Earth Observations and Remote Sensing.

[8] Inglada J (2015). Assessment of an Operational System for Crop Type Map Production Using High Temporal and Spatial Resolution Satellite Optical Imagery. Remote Sensing.

[9] Fu Y (2021). A Satellite-Based Method for National Winter Wheat Yield Estimating in China. Remote Sensing.

[10] Song Y, Wang J (2019). Mapping Winter Wheat Planting Area and Monitoring Its Phenology Using Sentinel-1 Backscatter Time Series. Remote Sensing.

[11] Niu Q (2022). A 30&thinsp;m annual maize phenology dataset from 1985 to 2020 in China. Earth System Science Data.

[12] Chu L, Jiang C, Wang T, Li Z, Cai C (2021). Mapping and forecasting of rice cropping systems in central China using multiple data sources and phenology-based time-series similarity measurement. Advances in Space Research.

[13] Northrup DL, Basso B, Wang MQ, Morgan CLS, Benfey PN (2021). Novel technologies for emission reduction complement conservation agriculture to achieve negative emissions from row-crop production. Proc. Natl. Acad. Sci. USA.

[14] Ma M (2022). Development of a Process-Based N2O Emission Model for Natural Forest and Grassland Ecosystems. Journal of Advances in Modeling Earth Systems.

[15] Crane-Droesch A (2018). Machine learning methods for crop yield prediction and climate change impact assessment in agriculture. Environ. Res. Lett..

[16] Mohammadi A, Khoshnevisan B, Venkatesh G, Eskandari S (2020). A Critical Review on Advancement and Challenges of Biochar Application in Paddy Fields: Environmental and Life Cycle Cost. Analysis. Processes.

[17] DeLucia EH (2014). The Theoretical Limit to Plant Productivity. Environ. Sci. Technol..

[18] Yuan W (2016). Estimating crop yield using a satellite-based light use efficiency model. Ecological Indicators.

[19] Yuan W (2014). Multiyear precipitation reduction strongly decreases carbon uptake over northern China. Journal of Geophysical Research: Biogeosciences.

[20] Liu Y, Zhang J, Qin Y (2020). How global warming alters future maize yield and water use efficiency in China. Technological Forecasting and Social Change.

[21] Li E, Zhao J, Pullens JWM, Yang X (2022). The compound effects of drought and high temperature stresses will be the main constraints on maize yield in Northeast China. Science of The Total Environment.

[22] Shen, R. *et al*. A 30 m Resolution Distribution Map of Maize for China Based on Landsat and Sentinel Images. *Journal of Remote Sensing* **2022** (2022).

[23] Wu J (2021). Impact of climate change on maize yield in China from 1979 to 2016. Journal of Integrative Agriculture.

[24] Yuan W (2018). Opportunistic Market-Driven Regional Shifts of Cropping Practices Reduce Food Production Capacity of China. Earth’s Future.

[25] Inglada J, Vincent A, Arias M, Marais-Sicre C (2016). Improved Early Crop Type Identification By Joint Use of High Temporal Resolution SAR And Optical Image Time Series. Remote Sensing.

[26] Zhang S (2019). Developing a Method to Estimate Maize Area in North and Northeast of China Combining Crop Phenology Information and Time-Series MODIS EVI. IEEE Access.

[27] Luo Y, Zhang Z, Chen Y, Li Z, Tao F (2020). ChinaCropPhen1km: a high-resolution crop phenological dataset for three staple crops in China during 2000–2015 based on leaf area index (LAI) products. Earth System Science Data.

[28] Yan J (2016). Drivers of cropland abandonment in mountainous areas: A household decision model on farming scale in Southwest China. Land Use Policy.

[29] Wang S, Li J, Jin R (2016). Generalized Synchronization of Fractional Order Chaotic Systems with Time-Delay. International Journal of Mechanical Engineering and Applications.

[30] Lu H, Xie H, He Y, Wu Z, Zhang X (2018). Assessing the impacts of land fragmentation and plot size on yields and costs: A translog production model and cost function approach. Agricultural Systems.

[31] Liu W (2018). A sub-pixel method for estimating planting fraction of paddy rice in Northeast China. Remote Sensing of Environment.

[32] Zhang B, Kong X (2018). Land use system change and coupling coordination degree in China in recent 30 years based on fragmentation. Journal of Beijing Normal University (Natural Science) (in Chinese).

[33] You N (2021). The 10-m crop type maps in Northeast China during 2017–2019. Sci Data.

[34] Zhou Y (2019). Are There Sufficient Landsat Observations for Retrospective and Continuous Monitoring of Land Cover Changes in China?. Remote Sensing.

[35] Roy DP (2008). Multi-temporal MODIS–Landsat data fusion for relative radiometric normalization, gap filling, and prediction of Landsat data. Remote Sensing of Environment.

[36] Gao F (2017). Toward mapping crop progress at field scales through fusion of Landsat and MODIS imagery. Remote Sensing of Environment.

[37] Pott LP, Amado TJC, Schwalbert RA, Corassa GM, Ciampitti IA (2021). Satellite-based data fusion crop type classification and mapping in Rio Grande do Sul, Brazil. ISPRS Journal of Photogrammetry and Remote Sensing.

[38] Liu Q, Zhang S, Wang N, Ming Y, Huang C (2022). Fusing Landsat-8, Sentinel-1, and Sentinel-2 Data for River Water Mapping Using Multidimensional Weighted Fusion Method. IEEE Transactions on Geoscience and Remote Sensing.

[39] Guan, X. *et al*. Fusing MODIS and AVHRR products to generate a global 1-km continuous NDVI time series covering four decades. *Earth System Science Data Discussions* 1–32, 10.5194/essd-2021-156 (2021).

[40] Shen H (2022). A Spatiotemporal Constrained Machine Learning Method for OCO-2 Solar-Induced Chlorophyll Fluorescence (SIF) Reconstruction. IEEE Transactions on Geoscience and Remote Sensing.

[41] Yin Q, Liu M, Cheng J, Ke Y, Chen X (2019). Mapping Paddy Rice Planting Area in Northeastern China Using Spatiotemporal Data Fusion and Phenology-Based Method. Remote Sensing.

[42] Ding M (2020). Phenology-Based Rice Paddy Mapping Using Multi-Source Satellite Imagery and a Fusion Algorithm Applied to the Poyang Lake Plain, Southern China. Remote Sensing.

[43] Li X, Peng Q, Yuan W (2023). National Ecosystem Data Bank.

[44] Maus V (2016). A Time-Weighted Dynamic Time Warping Method for Land-Use and Land-Cover Mapping. IEEE Journal of Selected Topics in Applied Earth Observations and Remote Sensing.

[45] Dong J (2020). Early-season mapping of winter wheat in China based on Landsat and Sentinel images. Earth System Science Data.

[46] Belgiu M, Csillik O (2018). Sentinel-2 cropland mapping using pixel-based and object-based time-weighted dynamic time warping analysis. Remote Sensing of Environment.

[47] Peng Q (2023). Science Data Bank.

[48] Tahsin S, Medeiros SC, Hooshyar M, Singh A (2017). Optical Cloud Pixel Recovery via Machine Learning. Remote Sensing.

[49] Vintrou E (2012). Crop area mapping in West Africa using landscape stratification of MODIS time series and comparison with existing global land products. International Journal of Applied Earth Observation and Geoinformation.

[50] Waldner F (2016). Towards a set of agrosystem-specific cropland mapping methods to address the global cropland diversity. International Journal of Remote Sensing.

[51] Cai Z (2022). An Adaptive Image Segmentation Method with Automatic Selection of Optimal Scale for Extracting Cropland Parcels in Smallholder Farming Systems. Remote Sensing.

[52] Zhang Q, Yuan Q, Zeng C, Li X, Wei Y (2018). Missing Data Reconstruction in Remote Sensing Image With a Unified Spatial–Temporal–Spectral Deep Convolutional Neural Network. IEEE Transactions on Geoscience and Remote Sensing.

[53] Xiao C, Li P, Feng Z, Wu X (2018). Spatio-temporal differences in cloud cover of Landsat-8 OLI observations across China during 2013–2016. J. Geogr. Sci..

[54] Forkuor G, Dimobe K, Serme I, Tondoh JE (2018). Landsat-8 vs. Sentinel-2: examining the added value of sentinel-2′s red-edge bands to land-use and land-cover mapping in Burkina Faso. GIScience & Remote Sensing.

[55] Kang Y, Meng Q, Liu M, Zou Y, Wang X (2021). Crop Classification Based on Red Edge Features Analysis of GF-6 WFV Data. Sensors.

[56] Peng, Q. *et al*. A new method for classifying maize by combining the phenological information of multiple satellite-based spectral bands. *Frontiers in Environmental Science* **10** (2023).

