# Supplementary material for: A twenty-year dataset of high-resolution maize distribution in China
Source: Sci Data. 2023 Sep 26;10:658. doi: 10.1038/s41597-023-02573-6 (PMC10522722; doi:10.1038/s41597-023-02573-6)
Supplement: Supplementary file 1 — Supplementary information [file 41597_2023_2573_MOESM1_ESM.pdf]

Table S1: Detailed confusion matrices of the distribution map of maize in 22 provinces. The rows in the confusion matrices mean the number of field identified samples, and the columns mean the number of surveyed samples.

Province	Year	Class	Maize	Other	UA (%)	PA (%)	OA (%)
Anhui	2012	Maize	413	65	74.55	86.40	79.96
		Other	141	409	86.29	74.36	
	2019	Maize	190	31	57.93	85.97	75.18
		Other	138	322	91.22	70.00	
Chongqing	2019	Maize	12	0	24.00	100.00	64.81
		Other	38	58	100.00	60.42	
Gansu	2008	Maize	76	75	100.00	50.33	73.78
		Other	0	135	64.29	100.00	
	2019	Maize	114	7	74.51	94.21	84.82
		Other	39	143	95.33	78.57	
Guangxi	2019	Maize	13	6	26.00	68.42	66.41
		Other	37	72	92.31	66.06	
Guizhou	2019	Maize	34	25	64.15	57.63	73.65
		Other	19	89	78.07	82.41	
Hebei	2007	Maize	1328	39	86.12	97.15	92.20
		Other	214	1662	97.71	88.59	
	2019	Maize	809	117	80.18	87.37	84.25
		Other	200	887	88.35	81.60	
Heilongjiang	2002	Maize	469	28	72.38	94.37	90.52
		Other	179	1508	98.18	89.39	
	2007	Maize	254	51	34.32	83.28	75.90
		Other	486	1437	96.57	74.73	
	2019	Maize	198	51	81.82	79.52	84.14
		Other	44	306	85.71	87.43	
Henan	2002	Maize	383	98	42.18	79.63	78.16
		Other	525	1847	94.96	77.87	
	2013	Maize	144	248	47.52	36.73	53.06
		Other	159	316	56.03	66.53	
	2019	Maize	618	198	73.57	75.74	82.72
		Other	222	1393	87.55	86.25	
Hubei	2012	Maize	96	69	64.43	58.18	88.60
		Other	53	852	92.51	94.14	
	2019	Maize	62	30	31.63	67.39	62.12
		Other	134	207	87.34	60.70	
Hunan	2019	Maize	9	0	27.27	100.00	76.24
		Other	24	68	100.00	73.91	
Inner Mongolia	2011	Maize	1544	182	70.53	89.46	78.78
		Other	645	1527	89.35	70.30	
	2019	Maize	212	55	89.45	79.40	86.23

		Other	25	289	84.01	92.04	
Jiangsu	2019	Maize	168	41	82.35	80.38	81.88
		Other	36	180	81.45	83.33	
Jilin	2002	Maize	737	20	84.13	97.36	94.38
		Other	139	1934	98.98	93.29	
	2019	Maize	74	16	88.10	82.22	90.44
		Other	10	172	91.49	94.51	
Liaoning	2013	Maize	1592	6	83.66	99.62	91.51
		Other	311	1823	99.67	85.43	
	2019	Maize	314	22	79.70	93.45	91.77
		Other	80	824	97.40	91.15	
Ningxia	2012	Maize	463	475	92.97	49.36	68.83
		Other	35	663	58.26	94.99	
	2019	Maize	129	23	80.63	84.87	84.79
		Other	31	172	88.21	84.73	
Shaanxi	2012	Maize	84	48	81.55	63.64	90.69
		Other	19	569	92.22	96.77	
	2019	Maize	243	47	92.05	83.79	86.59
		Other	21	196	80.66	90.32	
Shandong	2012	Maize	3743	60	87.41	98.42	89.88
		Other	539	1578	96.34	74.54	
	2019	Maize	1094	444	71.41	71.13	87.48
		Other	438	5067	91.94	92.04	
Shanxi	2002	Maize	374	134	85.39	73.62	89.05
		Other	64	1237	90.23	95.08	
	2019	Maize	111	41	78.17	73.03	76.92
		Other	31	129	75.88	80.63	
Sichuan	2013	Maize	18	8	40.91	69.23	79.88
		Other	26	117	93.60	81.82	
	2019	Maize	58	22	71.60	72.50	76.06
		Other	23	85	79.44	78.70	
Tianjin	2019	Maize	74	34	88.10	68.52	76.72
		Other	10	71	67.62	87.65	
Xinjiang	2019	Maize	69	21	54.33	76.67	75.16
		Other	58	170	89.01	74.56	
Yunnan	2005	Maize	226	0	46.79	100.00	79.46
		Other	257	768	100.00	74.93	
	2019	Maize	44	9	61.97	83.02	77.91
		Other	27	83	90.22	75.45	
